# Transcatheter aortic valve replacement- management of patients with significant coronary artery disease undergoing aortic valve interventions: surgical compared to catheter-based approaches in hybrid procedures

**DOI:** 10.1186/s12872-019-1087-2

**Published:** 2019-05-14

**Authors:** Hardy Baumbach, Eva R. Schairer, Kristina Wachter, Christian Rustenbach, Samir Ahad, Alina Stan, Stephan Hill, Peter Bramlage, Ulrich F. W. Franke, Tim Schäufele

**Affiliations:** 1 Department of Cardiovascular Surgery, Robert Bosch Hospital, Auerbachstr. 110, 70376 Stuttgart, Germany; 2 Department of Cardiology, Robert Bosch Hospital, Stuttgart, Germany; 3 Cardiothoracic Surgery, Heartcenter University Hospital Cologne, Cologne, Germany; 4 Institute for Pharmacology and Preventive Medicine, Cloppenburg, Germany

**Keywords:** Aortic stenosis, Coronary artery disease, Aortic valve replacement, Off-pump coronary artery bypass, Percutaneous coronary intervention

## Abstract

**Background:**

Coronary artery disease (CAD) is associated with poorer outcomes after aortic valve replacement (AVR). For high-risk patients with complex CAD, combined transcatheter aortic valve replacement (TAVR) plus off-pump/minimally-invasive coronary artery bypass (OPCAB/MIDCAB) has been proposed.

**Methods:**

A prospective registry analysis was performed to compare the characteristics and outcomes of patients undergoing TAVR+OP/MIDCAB with those undergoing TAVR plus percutaneous coronary intervention (PCI) and surgical AVR plus coronary artery bypass grafting (CABG) between 2008 and 2015 at a single site in Germany.

**Results:**

464 patients underwent SAVR+CABG, 50 underwent TAVR+OP/MIDCAB, and 112 underwent TAVR+PCI. The mean ages (*p* < 0.001) and logistic EuroSCOREs (p < 0.001) were similarly higher in TAVR+OP/MIDCAB and TAVR+PCI patients compared to SAVR+CABG patients. Prior cardiac surgery was more common in TAVR+PCI than in TAVR+OP/MIDCAB and SAVR+CABG patients (p < 0.001). Procedural times were shortest (p < 0.001), creatine kinase (muscle brain) levels least elevated (p < 0.001), pericardial tamponade least common (*p* = 0.027), and length of hospital stay shortest (*p* = 0.011) in TAVR+PCI, followed by TAVR+OP/MIDCAB and SAVR+CABG patients. In-hospital mortality was highest for TAVR+OP/MIDCAB patients (18.0%) with comparable rates for TAVR+PCI and SAVR+CABG groups (9.0 and 6.9%; *p* = 0.009). Mortality by 12 months was more probable after TAVR+OP/MIDCAB (HR: 2.17, *p* = 0.002) and TAVR/PCI (HR: 1.63, *p* = 0.010) than after SAVR+CABG, with the same true of rehospitalisation (HR: 2.39, *p* = 0.003 and HR: 1.63, *p* = 0.033).

**Conclusions:**

TAVR+OP/MIDCAB patients share many characteristics with TAVR+PCI patients, with only slightly poorer long-term outcomes. In patients ineligible for SAVR+CABG and TAVR+PCI, hybrid interventions are reasonable second-line options.

**Electronic supplementary material:**

The online version of this article (10.1186/s12872-019-1087-2) contains supplementary material, which is available to authorized users.

## Background

An estimated 40–75% of the severe aortic stenosis (AS) patients who undergo transcatheter aortic valve replacement (TAVR) have concomitant coronary artery disease (CAD) [1]. In patients with a primary indication for surgical aortic valve replacement (SAVR), a history of coronary artery bypass grafting (CABG) increases operative risk; to diminish this effect, guidelines recommend combination of both procedures into one hybrid operation [2]. For patients ineligible for SAVR, TAVR plus coronary revascularisation hybrids have now been suggested. Besides reducing the number of surgeries a patient must endure, revascularisation just prior to TAVR also minimises the risk of coronary ischaemia during rapid ventricular pacing, to which the hypertrophied myocardium is particularly vulnerable .

A number of studies have suggested the comparable safety and efficacy of TAVR plus PCI to that of isolated TAVR [3, 4]. Guidelines now tentatively recommend this combination for severe AS patients with concomitant coronary stenosis occupying > 70% of the artery diameter [2]; however not all coronary lesions are treatable with PCI. More recently, off-pump CABG (OPCAB) has been proposed as a method of complete coronary revascularisation in patients with complex stenosis and/or high SYNTAX scores [5, 6], with the off-pump technique avoiding the harmful effects of cardiopulmonary-bypass (CPB) [7]. OPCAB has been used to treat multi-vessel disease requiring a larger operative area, with a recent 4-patient series outlining its successful use immediately prior to transaortic (TAo) TAVR [8]. A modified version of OPCAB, known as minimally invasive direct CABG (MIDCAB), has been developed for the treatment of single-vessel disease, achievable through a smaller incision. Since the first documented MIDCAB plus transapical (TA) TAVR hybrid in 2010 [9], several other successful cases have been reported [5, 10]. However, only one larger study has evaluated the safety and feasibility of TAVR+OPCAB/MIDCAB to date [11], and no comparisons with TAVR+PCI and SAVR+CABG have been performed.

The aim of the present study was to compare the characteristics and outcomes of patients undergoing TAVR+OP/MIDCAB with those undergoing TAVR+PCI, with data for those undergoing SAVR+CABG included as a benchmark control. This information will add to the currently limited pool of evidence for each TAVR hybrid.

## Methods

The present prospective, observational single-center study was carried in Germany between January 2008 and October 2015. Elderly patients with severe AS and concomitant extensive CAD who had a primary indication for aortic valve replacement were consecutively enrolled. The study received prior approval from the “Ärztekammer Stuttgart” institutional review board and was carried out in accordance with the declaration of Helsinki. All patients provided their written informed consent.

### Inclusion/exclusion criteria

Severe AS patients with a primary indication (without contraindications) for AVR at our site were included. Those who elected not to undergo one of these procedures; who underwent concomitant procedures other than CABG, OPCAB, MIDCAB and PCI; or who had sclerosis rather than stenosis of coronary vessels were excluded. Other exclusion criteria included the iatrogenic suturing of a coronary ostium and the fact that the date of PCI was not within 12 months before TAVR. Eligible patients were divided into three groups depending upon hybrid intervention type (SAVR+CABG; TAVR+OPCAB or TAVR+MIDCAB [TAVR+OP/MIDCAB]; and TAVR+PCI).

### Choice of surgical vs. transcatheter aortic valve replacement

SAVR was the preferred AVR procedure, unless the patient was deemed ineligible by an interdisciplinary Heart Team based on factors such as a high surgical risk score, advanced age, relevant comorbidities, and short life expectancy. Such patients were evaluated for TAVR eligibility, with access route determined by a careful assessment of patient anatomy considered alongside the preferred coronary revascularisation approach (see below).

### Choice of coronary revascularisation approach

CAD complexity was evaluated in all patients with a primary indication for SAVR or TAVR, and those with an intermediate/high Synergy between PCI with Taxus and Cardiac Surgery (SYNTAX) score [12] were indicated for concomitant revascularisation. For patients scheduled to undergo SAVR, CABG through open surgery was the preferred revascularisation technique. For those scheduled for TAVR, the mode of revascularisation was determined by thorough assessment of the number and complexity of diseased coronary vessels. Unless considered unsuitable due to left anterior descending artery (LAD) proximal lesions or an extremely high SYNTAX score, patients preferentially underwent PCI, with the TAVR access route determined by the Heart Team. In the case of single-vessel LAD or left circumflex artery disease, MIDCAB via a left antero-lateral minithoracotomy followed by TA-TAVR was preferred. In the case of multi-vessel complex disease, OPCAB via a median sternotomy followed by TAo-TAVR was preferred.

### Definitions

Pulmonary disease was defined as COPD with or without medication. CKD was defined as to KDIGO/KDOQI and considered compensated when CKD stabilizes on Stage III. Prior cardiac surgery included any surgical valve replacement or reconstruction or CABG or any other surgical intervention at the thoracic aorta. A history of myocardial infarction (MI) was considered present if MI had occurred within the last 90 days. A history of stroke/TIA was considered as an event happened during lifetime which is severely affecting day-to-day functioning. Hospitalisation during follow-up was divided into early (< 3 months after intervention) and late (> 3 months after intervention) hospitalisation and was defined as any event which lead to a hospital stay.

### Statistical analysis

Continuous variables are presented as means ± standard deviations (SD) or medians and interquartile ranges (IQR) and categorical variables as absolute numbers and frequencies (%). ANOVA, Kruskal-Wallis tests were used for comparisons across the three study groups, with t-test and Wilcoxon- Mann-Whitney-test used to compare the two TAVR groups only, where appropriate. Freedom from death and rehospitalisation were estimated using Kaplan-Meier analysis and a log-rank test. Cox proportional hazard coefficients and *p*-values (adjusted for age, EuroSCORE, left ventricular ejection fraction [LVEF], New York Heart Association [NYHA] class, pulmonary disease (asthma bronchiale, lung cancer, pulmonary fibrosis), pulmonary hypertension, arrhythmias, chronic kidney disease [CKD], aortic insufficiency, and mitral/tricuspid valve disease) were calculated for each of the TAVR hybrids. Effective orifice area (EOA), body mass index (BMI) and mean aortic valve (AV) gradient were not adjusted for, owing to a high number of missing values. All analyses were carried out using SPSS version 24.0 (IBM Corporation, Armonk, NY, USA), with *p*-values of < 0.05 considered significant.

## Results

**Fig. 1:**
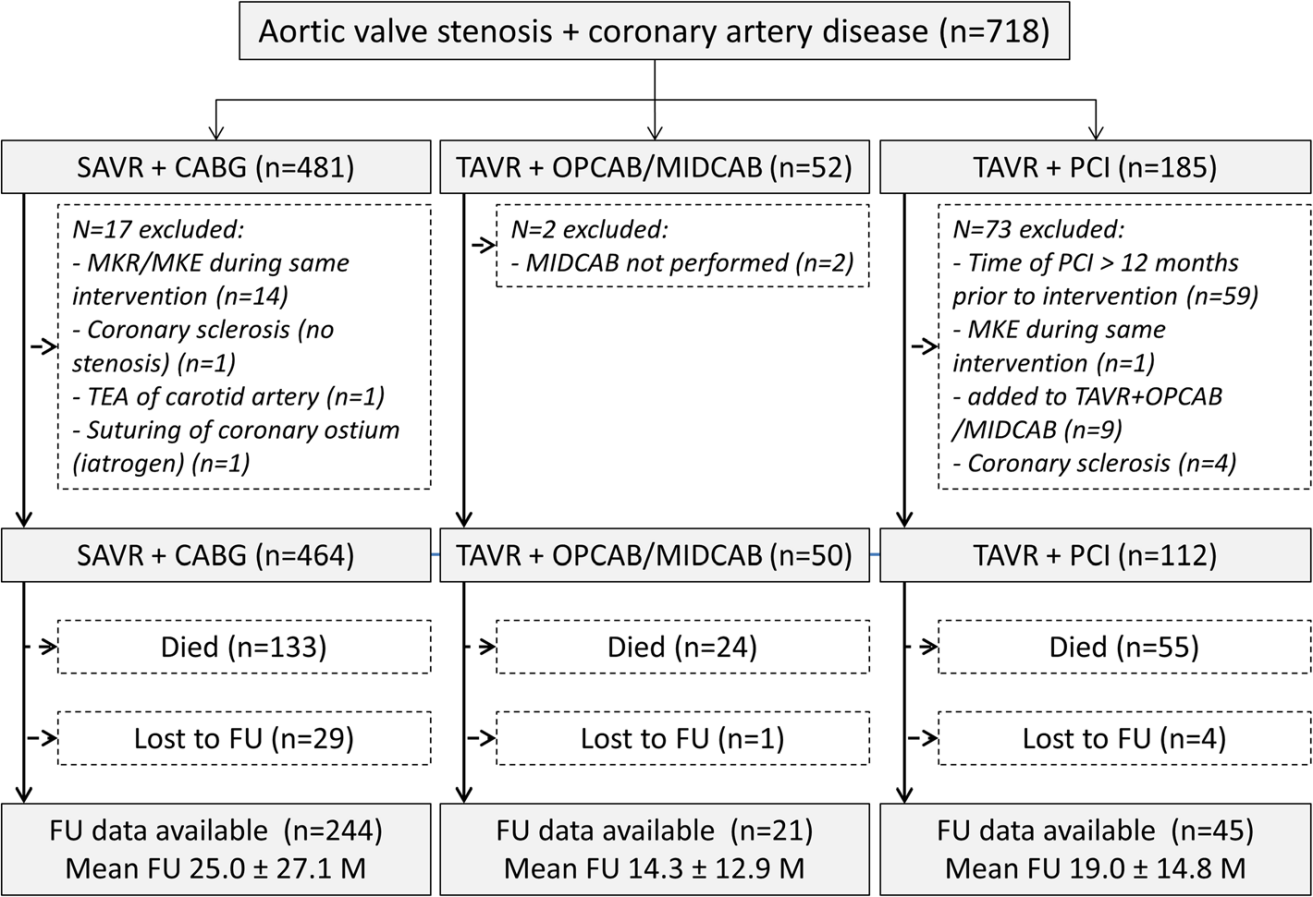
Patient flow

Of the 718 patients initially enrolled, concomitant coronary revascularisation and aortic valve replacement was performed in 626 cases (87.2%) (Fig. 1). Of these, 464 patients (74.1%) underwent SAVR+CABG, 50 (8.0%) underwent TAVR+OP/MIDCAB as hybrid procedures (for details see below), and 112 (17.9%) underwent TAVR+PCI. Of the 50 patients undergoing TAVR+OP/MIDCAB 24 patients underwent OPCAB and 26 MIDCAB.

### Patient characteristics

The mean ages of TAVR+OP/MIDCAB and TAVR+PCI patients were similar, both being higher than in the SAVR+CABG group (82.1 and 81.3 vs. 78.7 years; *p* < 0.001) (Table 1). The same trend was evident for the prevalence of pulmonary hypertension, compensated CKD, prior dialysis, and tricuspid valve disorders, as well as mean logistic EuroSCORE (36.4 and 36.1 vs. 21.0; p < 0.001). NYHA class III/IV was most common in the TAVR+PCI group, followed by the TAVR+OP/MIDCAB and SAVR+CABG groups (p < 0.001), with the same trend seen for pulmonary and mitral valve disease. Prior cardiac surgery was much more common in the TAVR+PCI compared to TAVR+OP/MIDCAB and SAVR+CABG groups (33.9% vs. 2.0 and 4.5%; *p* < 0.001). TAVR+PCI and TAVR+OP/MIDCAB patients had comparable mean EOAs and LVEFs, significantly lower compared to SAVR+CABG patients. The mean AV gradient was slightly higher in the TAVR+PCI than in the TAVR+OP/MIDCAB and SAVR+CABG groups.

**Table 1:** Baseline patient characteristics

	SAVR + CABG	TAVR + OP/MIDCAB	TAVR + PCI	*p*-value (across groups)
	(*n* = 464)	(*n* = 50)	(*n* = 112)	
	*mean ± SD or median (IQR) or n (%)*	*mean ± SD or median (IQR) or n (%)*	*mean ± SD or median (IQR) or n (%)*	
Age (years)	78.7 ± 3.1	82.1 ± 4.3	81.3 ± 5.7	< 0.001
Female gender	168 (36.2)	19 (38.0)	39 (34.8)	0.923
BMI (kg/m^2^)	26.2 ± 3.8	26.1 ± 4.7	26.0 ± 3.9	0.011
Clinical history				
History of stroke/TIA	46 (9.9)	7 (14.0)	16 (14.3)	0.675
History of MI (< 90 days)	138 (29.8)	28 (56.0)	79 (70.5)	0.318
Previous cardiac surgery	21 (4.5)	1 (2.0)	38 (33.9)	< 0.001
Diabetes mellitus	180 (38.8)	19 (38.0)	33 (29.5)	0.318
Hypertension	454 (98.1)	50 (100.0)	109 (97.3)	0.516
Pulmonary disease	56 (12.1)	9 (18.0)	27 (24.1)	0.020
Pulmonary hypertension^a^	128 (27.6)	27 (54.0)	62 (55.4)	< 0.001
Chronic kidney disease				
Compensated	115 (24.8)	22 (44.0)	49 (43.8)	< 0.001
Prior dialysis	8 (1.7)	3 (6.0)	7 (6.3)	0.014
Other valve disorders				
Mitral	235 (50.6)	32 (64.0)	86 (76.8)	< 0.001
Tricuspid	17 (3.7)	6 (12.0)	12 (10.7)	< 0.001
EuroSCORE I (%)	21.0 ± 17.5	36.4 ± 22.4	36.1 ± 18.9	< 0.001
NYHA class				< 0.001
I/II	206 (44.4)	8 (16.0)	10 (8.9)	
III/IV	258 (55.6)	42 (84.0)	102 (91.1)	
Multi-vessel CAD	359 (77.4)	42 (84.0)	83 (74.1)	0.570
AV parameters				
LVEF (%)	55.7 ± 13.8	48.3 ± 14.4	51.3 ± 2.6	< 0.001
EOA (cm^2^)	0.8 ± 0.3	0.7 ± 0.2	0.7 ± 0.2	< 0.001
Mean AV gradient (mmHg)	40.7 ± 17.1	40.4 ± 14.0	42.5 ± 15.2	0.027
Peak AV gradient (mmHg)	66.6 ± 25.7	67.9 ± 19.7	72.2 ± 23.3	0.150
Annulus size (cm^2^)	22.9 ± 4.2	23.7 ± 2.2	22.9 ± 2.4	0.092

*Legend:* ^a^PapSys > 30 mmHg

### Peri-procedural details

The TA route was most commonly used in both the TAVR+PCI (62.5%) and TAVR+OP/MIDCAP (58.0%) patients (Table 2). However, while the majority of the remaining TAVR+PCI patients underwent TF-TAVR (36.6%; *p* < 0.001), all of the remaining TAVR+OP/MIDCAB patients underwent TAo-TAVR.

**Table 2:** Peri-procedural details and hospital stay

	SAVR + CABG	TAVR + OP/MIDCAB	TAVR + PCI	p-value (across groups)
	(n = 464)	(n = 50)	(n = 112)	
	*mean ± SD or median (IQR) or n (%)*	*mean ± SD or median (IQR) or n (%)*	*mean ± SD or median (IQR) or n (%)*	
Access route				< 0.001
Transfemoral	n.a.	0 (0.0)	41 (36.6)	
Transapical	n.a.	29 (58.0)	70 (62.5)	
Transaortic	n.a.	21 (42.0)	1 (0.9)	
Prosthesis diameter (mm)	23.7 ± 2.3	26.1 ± 2.1	25.8 ± 2.1	< 0.001
	23 (23–25)	26 (26–29)	26 (23–27)	
Total intervention time (min)	221 (190–245)	175 (146–231)	67 (53–83)	< 0.001
Total length of hospital stay (d)	12.0 (9.0–17.0)	11.0 (7.0–20.0)	10.0 (7.0–15.0)	0.011
Length of ICU stay (d)	1.0 (1.0–4.0)	1.0 (1.0–4.0)	1.0 (1.0–1.0)	0.001
Erythrocyte packs/patient	2.7 ± 3.9	3.6 ± 4.29	1.7 ± 2.9	0.001

Every patient in the TAVR+PCI group underwent two separate procedures (PCI first, TAVR as a second procedure, however not more than 12 months later). Every patient in the TAVR+OP/MIDCAB- group had their intervention in one procedure.

The median procedural duration was shortest for TAVR+PCI patients, followed by TAVR+OP/MIDCAB and SAVR+CABG patients (67, 175, and 221 min; p < 0.001). The lowest number of erythrocyte packs were used in the TAVR+PCI, followed by the SAVR+CABG and TAVR+OP/MIDCAB groups (*p* = 0.001).

### Complications

Conversion to open surgery was necessary in three TAVR+PCI patients (2.7%: all due to grade III aortic insufficiency) and two TAVR+OP/MIDCAB patients (4.0%: one due to intraoperative prosthesis dislocation and one due to resuscitation) (Table 3). Most cases of re-thoracotomy became necessary due to tamponade or bleeding complications. No case is known where a re-thoracotomy had to be performed because of bypass or stent insufficiency. Only one peri-procedural death occurred (TAVR+PCI patient with grade III aortic insufficiency) because of ventricular rupture. The rates of intra-operative (within 72 h) stroke/TIA and MI were low and not significantly different across groups; however, creatine kinase (muscle brain type [CK-MB]) levels were least elevated in TAVR+PCI patients, followed by TAVR+OP/MIDCAB and SAVR+CABG groups (31.1 ± 35.0, 47.2 ± 81.8 and 51.1 ± 57.1 U/l; *p* < 0.001). The rate of pericardial tamponade followed a similar trend (0.9, 2.0 and 6.7%; *p* = 0.027). Conversely, intra-operative resuscitation was required in only 0.4% of SAVR+CABG patients, compared to 2.7 and 4.0% of TAVR+PCI and TAVR+OP/MIDCAB patients, respectively (*p* = 0.017).

**Table 3:** Peri-procedural and post-operative complications

	SAVR + CABG	TAVR + OP/MIDCAB	TAVR + PCI	p-value (across groups)
	(n = 464)	(n = 50)	(n = 112)	
	*mean ± SD or median (IQR) or n (%)*	*mean ± SD or median (IQR) or n (%)*	*mean ± SD or median (IQR) or n (%)*	
Intra-operative mortality	0 (0.0)	0 (0.0)	1 (0.9)	0.009
Intra-operative resuscitation	2 (0.4)	2 (4.0)	3 (2.7)	0.017
Myocardial infarction	2 (0.4)	1 (2.0)	0 (0.0)	0.225
CK-MB (U/l)	51.1 ± 57.1	47.2 ± 81.8	31.1 ± 35.0	< 0.001
Stroke/TIA	23 (5.0)	1 (2.0)	5 (4.5)	0.622
Conversion to open surgery^a^	n.a.	2 (4.0)^b^	3 (2.7)^c^	0.017
Re-thoracotomy	36 (7.8)	5 (10.0)	3 (2.7)	0.117
Pericardial tamponade	31 (6.7)	1 (2.0)	1 (0.9)	0.027
AKI stage II/III	48 (10.3)	5 (10.0)	11 (9.8)	0.984
Post-op. resuscitation (30d)	34 (7.3)	5 (10.0)	6 (5.4)	0.557
Post-operative dialysis (30d)	39 (8.4)	5 (10.0)	14 (12.5)	0.190
Permanent	5 (1.1)	2 (4.0)	5 (4.5)	0.028
Post-operative AF (30d)	41 (8.8)	3 (6.0)	4 (3.6)	0.154
Post-operative PPI (30d)	25 (5.4)	1 (2.0)	11 (9.9)	0.091
In-hospital mortality	32 (6.9)	9 (18.0)	9 (9.0)	0.009
30d overall mortality	34 (7.4)	8 (16.0)	7 (6.3)	0.077

*Legend:* ^a^Conversion to open surgery was defined as sternotomy and change to SAVR with a heart-lung-machine,^b^one patient experienced intraoperative dislocation of the prosthesis, the second patient was resuscitated; ^c^all due to severe aortic regurgitation, one patient died as a result

Post-operatively, a smaller proportion of SAVR+CABG patients required permanent dialysis compared to TAVR+OP/MIDCAB and TAVR+PCI patients (1.1% vs. 4.0 and 4.5%; *p* = 0.028). In-hospital mortality was highest for TAVR+OP/MIDCAB patients with rates comparable between TAVR+PCI and SAVR+CABG patients (18.0% vs. 9.0 and 6.9%; *p* = 0.009). Of the 9 patients (18%) in the TAVR+OP/MIDCAB group who died in hospital, 4 died from acute circulatory collapse with cardiogenic shock, 2 due to multi organ failure following acute myocardial infarction and 3 patients died due to no cardiac cause. In the TAVR+PCI group 9 (9%) died, 3 of them due to acute circulatory collapse with cardiogenic shock, 1 patient suffered a ventricular rupture and another patient a stroke following multi organ failure. 4 patients died due to no cardiac cause.

No patient undergoing the TAVR+MIDCAB procedure underwent additional PCI of a right-sided target vessel following the index procedure.

### Hospital stay

The total length of stay in hospital following the intervention was shortest for patients who underwent TAVR+PCI, followed by TAVR+OP/MIDCAB and SAVR+CABG (10.0 [7.0–15.0], 11.0 [7.0–20.0], and 12.0 [9.0–17.0] days; *p* = 0.011) (Table 2). During this time, a median of one day was spent in ICU for all groups.

### Follow-up

Follow-up data were available for 45 TAVR+PCI patients (40.2%), 21 TAVR+OP/MIDCAB patients (42%) and 247 SAVR+CABG patients (53.2%). Mean follow-up times were 19.0 ± 14.8 months, 14.3 ± 12.9 months, and 25.0 ± 27.1 months, respectively.

**Fig. 2:**
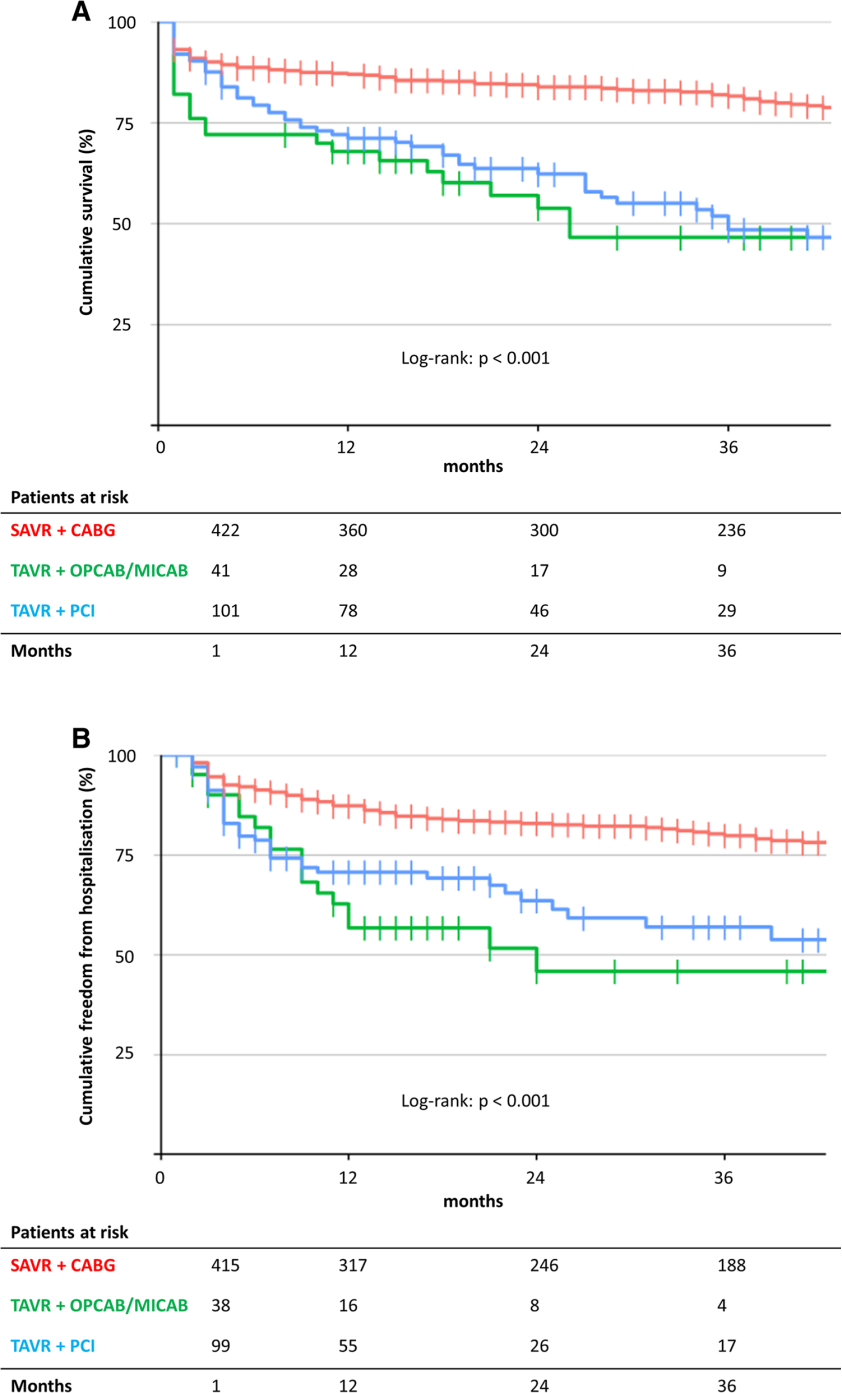
Kaplan-Meier analysis for **a**) mortality and **b**) any rehospitalisation

At 12 months, survival rate was poorest in the TACI+OP/MIDCAB (65.5%), followed by the TAVR+PCI (71.1%) and SAVR+CABG (86.7%; log-rank test: *p* < 0.001) group (Fig. 2a). The same was true of rehospitalisation (53.6, 69.0, and 86.6%, respectively; log-rank test: p < 0.001) (Fig. 2b). Adjusted Cox hazard regression revealed that the likelihood of mortality by 12 months was higher in patients undergoing TAVR+OP/MIDCAB (HR: 2.17; 95% CI: 6.98–43.02; *p* = 0.002) and TAVR/PCI (HR: 1.63, 95% CI: 13.36–56.66; *p* = 0.010) procedures. A similar trend was observed for any rehospitalisation outcome (HR: 2.39, 95% CI: 19.84–34.46; *p* = 0.003 and HR: 1.63, 95% CI: 23.40–88.62; *p* = 0.033, respectively).

### Hybrid procedures

Of the 50 patients that underwent a hybrid procedure, 24 underwent TAVR+OPCAB and 26 TAVI+MIDCAB. Although these groups were very small compared to the principal comparison groups, we explored these further in Additional file 1: Table S1 and Additional file 2 Table S2, as well as in Additional file 3: Figure S1 and Additional file 4: Figure S2.

## Discussion

While the characteristics of patients undergoing TAVR+OP/MIDCAB and TAVR+PCI were fairly similar, those with a history of cardiac surgery appeared more likely to be managed with TAVR+PCI. Both TAVR hybrids resulted in less myocardial injury, less pericardial tamponade, shorter procedural durations, and shorter hospital stays compared to SAVR+CABG, with these advantages particularly marked for TAVR+PCI. While both TAVR hybrids were associated with a greater need for intra-operative resuscitation and permanent dialysis, as well as a decreased likelihood of survival and freedom from rehospitalisation at 12 months compared to SAVR+CABG, these outcomes were not vastly dissimilar between the two TAVR groups.

### Complications

All three hybrid procedures had excellent peri-procedural safety, with low and comparable rates of intra-operative death, stroke/TIA and MI across groups. However, CK-MB, a marker of myocardial injury, was significantly less elevated in patients undergoing TAVR hybrids compared to SAVR+CABG. A greater rise in CK-MB levels has been associated with increased cardiovascular mortality and poorer clinical outcomes [13]. This highlights the importance of this finding, which is supported by a previous study of isolated aortic valve replacement procedures that observed a smaller rise in markers of cardiac injury after TAVR compared to SAVR [14]. This benefit may be explained by the avoidance of CPB in TAVR hybrids. Furthermore, procedural duration, a known predictor of post-TAVR myocardial injury [15], was longer in the TAVR+OP/MIDCAB than TAVR+PCI group and may explain the lower CK-MB levels in the latter patients. Nevertheless, for patients with complex CAD in whom TAVR+PCI is not feasible, use of TAVR+OP/MIDCAB appears to result in an acceptable degree of myocardial injury, with length of surgery expected to decrease with experience.

Pericardial tamponade was much less common in the TAVR hybrid groups than in the SAVR+CABG group, likely reflecting the shorter, less invasive procedures requiring a lesser degree of heart manipulation. Indeed, prolonged intervention time has been identified as a risk factor for pericardial effusion during cardiac surgery [16]. The same logic may be applied to the lower rate of pericardial tamponade in the TAVR+PCI compared to TAVR+OP/MIDCAB patients. The high number of patients with prior cardiac surgery in the TAVR+PCI group may also have relevance here, given that previous cardiac operations have been associated with a lower risk of effusion [16]. Conversion to open surgery was required in a slightly smaller proportion of TAVR+PCI patients compared to TAVR+OP/MIDCAB patients. The reason for this is unclear, though is more likely to be related to TAVR access route rather than to revascularisation technique, given that the reasons for conversion were mainly TAVR complications.

The rate of in-hospital mortality (18%) was particularly high for TAVR+OP/MIDCAB patients. This is likely a combination of their significantly higher surgical risk (EuroSCORE 36.4%), disease burden, and age compared to the SAVR+CABG group, and the fact that the procedure was more invasive and involved compared to TAVR+PCI. Indeed, a mortality rate of 18.8% has been reported for patients with a EuroSCORE of 33.8% undergoing isolated TAVR involving a mini-thoracotomy [17], which is similar to that of the current composite procedure. Another possible contributor is learning curve, given that the TAVR+OP/MIDCAB hybrid is relatively new and infrequently performed, particularly in the case of MIDCAB. Thus, poorer survival rates are to be expected. However, when compared to the 14.3% 30-day mortality rate previously reported for this particular hybrid procedure in a comparable patient population [11], the rate in the present study is still particularly elevated. The reason for this remains unclear, but is likely related to patient factors.

### Length of procedure and hospital stay

Due to different degrees of invasiveness, shorter procedural times are a known advantage of TAVR (particularly TF-TAVR) compared to SAVR [18], of PCI compared to OP/MIDCAB, and of MIDCAP compared to CABG [19]. It is therefore unsurprising that TAVR+PCI was the shortest procedure, followed by TAVR+OP/MIDCAB and SAVR+CABG, with the number of erythrocyte packs required following this trend. Furthermore, despite their higher risk profile, patients undergoing TAVR hybrids had shorter hospital stays than those who underwent SAVR+CABG, further indicating the potential for less invasive surgery to reduce resource utilisation. Indeed, reduced bed costs after TAVR have been identified as a major driver of its cost-effectiveness relative to SAVR [20]. Considering that an advantage of minimally-invasive surgery is its facility to allow earlier discharge, it is surprising that the length of hospital stay for TAVR+PCI patients was only moderately shorter than that for TAVR+OP/MIDCAB patients, despite nearly 40% of the former group undergoing the procedure via a purely percutaneous approach. However, this may be explained by the fact that baseline EuroSCORE and NYHA class, two factors identified as predictive for length of hospital stay after TAVR in clinical practice [18], were fairly comparable between TAVR groups. The ubiquitous use of general anaesthesia, despite PCI and TF-TAVR being feasible under conscious sedation, may also have contributed [21]. Nevertheless, there appears to be scope for further exploitation of early discharge after fully percutaneous hybrid procedures.

### Long-term survival and rehospitalisation

Long-term survival was poorer in both TAVR groups compared to the SAVR+CABG group. While risk factors such as a more advanced age, higher NYHA class, lower LVEF and a greater number of comorbidities were more prevalent in TAVR groups [22], a higher risk of mortality remained after adjustment for these and other possible confounding factors, the same being true of rehospitalisation. Thus, SAVR+CABG is likely to remain the gold standard in patients who are eligible. Though both the probability of death and rehospitalisation by 12 months were elevated to a greater degree in patients who underwent TAVR+OP/MIDCAB than in those who underwent TAVR+PCI, the differences were relatively small. Thus, TAVR+OP/MIDCAB may be considered a reasonable approach in patients who are ineligible for both SAVR+CABG and TAVR+PCI. Long-term direct comparisons between patients who undergo the hybrid procedure, those who undergo the two procedures separately, those who undergo one procedure only, and those who elect not to undergo either procedure would be interesting, but would require an extremely large dataset.

### Limitations

The main limitation of the present study was the lack of randomisation, resulting in different patient populations with varying n-numbers. However, this represents the real-world use of the different interventions, with TAVR reserved for higher-risk patients and OP/MIDCAB reserved for those in whom PCI is not feasible. A further limitation was the combination of TAVR+OPCAB and TAVR+MIDCAB patients into one group, considering that the procedures have important differences. However, small patient numbers prevented separate comparisons. As the procedures become more diffuse, larger comparative studies will be possible. Finally, follow-up data was available for only a small proportion of the study population; thus, comparisons regarding long-term mortality and rehospitalisation are severely limited and should be interpreted with caution.

## Conclusions

Patients undergoing TAVR+OP/MIDCAB share many characteristics with those undergoing TAVR+PCI. Both TAVR hybrids appear to result in shorter procedural durations, less complications and shorter length of hospital stay but mortality and rehospitalisation at 12 months are increased. Thus, in patients ineligible for SAVR+CABG and TAVR+PCI, hybrid interventions may be reasonable second-line options, especially if at high surgical risk and complex CAD.

## Additional files

Additional file 1: **Table S1.** Baseline patient characteristics TAVR+OP vs. MIDCAB. (DOCX 21 kb)

Additional file 2: **Table S2.** Peri-procedural details, hospital stay and complications TAVR+OP vs. MIDCAB (DOCX 20 kb)

Additional file 3: **Figure S1.** Patient flow TAVR+OP vs. MIDCAB (TIF 528 kb)

Additional file 4: **Figure S2.** Kaplan-Meier analysis for A) mortality and B) any rehospitalisation TAVR+OP vs. MIDCAB. (ZIP 461 kb)

## Abbreviations

ABR: Aortic valve replacement
AV: Aortic valve
BMI: Body mass index
CABG: Coronary artery bypass grafting
CAD: Coronary artery disease
CKD: Chronic kidney disease
CPB: CardioPulmonary-bypass
EOA: Effective orifice area
LAD: Left anterior descending
LVEF: Left ventricular ejection fraction
MIDCAB: Minimally-invasive coronary artery bypass
NYHA: New York heart association
OPCAB: Off-pump coronary artery bypass
PCI: Percutaneous coronary intervention
SAVR: Surgical aortic valve replacement
SYNTAX: Synergy between PCI with Taxus and cardiac surgery
TA: Transapical
TAo: Transaortic
TAVR: Transcatheter aortic valve replacement
