# Supplementary material for: Transcatheter aortic valve replacement- management of patients with significant coronary artery disease undergoing aortic valve interventions: surgical compared to catheter-based approaches in hybrid procedures
Source: BMC Cardiovasc Disord. 2019 May 14;19:108. doi: 10.1186/s12872-019-1087-2 (PMC6515676; doi:10.1186/s12872-019-1087-2)
Supplement: Supplementary file 1 — Table S1. Baseline patient characteristics TAVR+OP vs. MIDCAB. (DOCX 21 kb) [file 12872_2019_1087_MOESM1_ESM.docx]

**Table S1 – Baseline patient characteristics TAVR+OP vs. MIDCAB**

|  | **TAVR+OP/MIDCAB**  **(n = 50)**  *mean ± SD or median (IQR) or n (%)* | **TAVR+OP**  **(n = 24)**  *mean ± SD or median (IQR) or n (%)* | **MIDCAB**  **(n = 26)**  *mean ± SD or median (IQR) or n (%)* |
| --- | --- | --- | --- |
| Age (years) | 82.1 ± 4.3 | 81.5 ± 3.8 | 82.6 ± 4.6 |
| Female gender | 19 (38.0) | 12 ( (50.0) | 19 (38.0) |
| BMI (kg/m^2^) | 26.1 ± 4.7 | 25.5 ± 5.0 | 26.8 ± 4.4 |
| Clinical history |  |  |  |
| History of stroke/TIA | 7 (14.0) | 4 (6.0) | 3 (11.5) |
| History of MI (<90 days) | 16 (32.0) | 8 (33.3) | 8 (30.8) |
| Previous cardiac surgery | 1 (2.0) | 0 (0) | 1 (3.8) |
| Diabetes mellitus | 19 (38.0) | 11 (45.8) | 8 (30.8) |
| Hypertension | 50 (100.0) | 24 (100.0) | 26 (100.0) |
| Pulmonary disease | 9 (18.0) | 5 (20.8) | 4 (15.4) |
| Pulmonary hypertension* | 27 (54.0) | 10 (41.7) | 17 (65.4) |
| Chronic kidney disease |  |  |  |
| Compensated | 22 (44.0) | 9 (37.5) | 13 (50.0) |
| Prior dialysis | 3 (6.0) | 2 (8.3) | 2 (7.7) |
| Other valve disorders |  |  |  |
| Mitral | 32 (64.0) | 16 (66.6) | 16 (61.5) |
| Tricuspid | 20 (40.0) | 8 (33.3) | 12 (46.2) |
| EuroSCORE I (%) | 36.4 ± 22.4 | 36.6 ± 24.6 | 36.2 ± 20.7 |
| NYHA class |  |  |  |
| I/II | 8 (16.0) | 2 (8.3) | 6 (23.0) |
| III/IV | 42 (84.0) | 22 (91.7) | 20 (67.9) |
| Multi-vessel CAD | 42 (84.0) | 23 (95.8) | 19 (73.1) |
| AV parameters |  |  |  |
| LVEF (%) | 48.3 ± 14.4 | 50.4 ± 16.7 | 46.4 ± 11.9 |
| EOA (cm^2^) | 0.7 ± 0.2 | 0.7 ± 0.2 | 0.8 ± 0.2 |
| Mean AV gradient (mmHg) | 40.4 ± 14.0 | 38.0 ± 14.6 | 42.7 ± 13.3 |
| Peak AV gradient (mmHg) | 67.9 ± 19.7 | 66.0 ± 20.8 | 69.7 ± 18.8 |
| Annulus size (cm^2^) | 23.7 ± 2.2 | 23.3 ± 2.4 | 24.0 ± 2.0 |

*Legend:* *PapSys >30 mmHg.
