# Supplementary material for: Transcatheter aortic valve replacement- management of patients with significant coronary artery disease undergoing aortic valve interventions: surgical compared to catheter-based approaches in hybrid procedures
Source: BMC Cardiovasc Disord. 2019 May 14;19:108. doi: 10.1186/s12872-019-1087-2 (PMC6515676; doi:10.1186/s12872-019-1087-2)
Supplement: Supplementary file 2 — Table S2. Peri-procedural details, hospital stay and complications TAVR+OP vs. MIDCAB (DOCX 20 kb) [file 12872_2019_1087_MOESM2_ESM.docx]

**Table 2 – Peri-procedural details, hospital stay and complications TAVR+OP vs. MIDCAB**

|  | **TAVR+OP/MIDCAB**  **(n = 50)**  *mean ± SD or median (IQR) or n (%)* | **TAVR+OP**  **(n = 24)**  *mean ± SD or median (IQR) or n (%)* | **MIDCAB**  **(n = 26)**  *mean ± SD or median (IQR) or n (%)* |
| --- | --- | --- | --- |
| Access route |  |  |  |
| Transfemoral | 0 (0.0) | 0 (0.0) | 0 (0.0) |
| Transapical | 29 (58.0) | 4 (16.7) | 25 (96.2) |
| Transaortic | 21 (42.0) | 20 (83.3) | 1 (3.8) |
| Prosthesis diameter (mm) | 26.1 ± 2.1  26 (26–29) | 25.6 ± 2.4  23 (21.25-25) | 24.0 ± 2.0  24 (22-26) |
| Total intervention time (min) | 175 (146–231) | 203 (171.25-251) | 151.5 (131.25-186.25) |
| Total length of hospital stay (d) | 11.0 (7.0–20.0) | 14.0 (7.0-24.0) | 10.0 (7.0-17.0) |
| Length of ICU stay (d) | 1.0 (1.0–4.0) | 1.0 (1.0-5.0) | 1.0 (1.0-4.0) |
| Erythrocyte packs/patient | 3.6 ± 4.29 | 3.7 ± 4.2 | 3.5 ± 4.3 |
| Intra-operative mortality | 0 (0.0) | 0 (0.0) | 0 (0.0) |
| Intra-operative resuscitation | 2 (4.0) | 0 (0.0) | 2 (7.7) |
| Myocardial infarction | 1 (2.0) | 0 (0.0) | 1 (3.8) |
| CK-MB (U/l) | 47.2 ± 81.8 | 31.9 ± 28.6 | 60.7 ± 108.3 |
| Stroke/TIA | 1 (2.0) | 1 (4.2) | 0 (0.0) |
| Conversion to open surgery^†^ | 2 (4.0)* | 0 (0.0) | 2 (7.7) |
| Re-thoracotomy | 5 (10.0) | 3 (12.5) | 2 (7.7) |
| Pericardial tamponade | 1 (2.0) | 1 (4.2) | 0 (0.0) |
| AKI stage II/III | 5 (10.0) | 3 (12.5) | 2 (7.7) |
| Post-op. resuscitation (30d) | 5 (10.0) | 2 (8.3) | 3 (11.5) |
| Post-operative dialysis (30d) | 5 (10.0) | 2 (8.3) | 3 (11.5) |
| Permanent | 2 (4.0) | 0 (0.0) | 2 (7.7) |
| Post-operative AF (30d) | 3 (6.0) | 2 (8.3) | 1 (3.8) |
| Post-operative PPI (30d) | 1 (2.0) | 0 (0.0) | 1 (3.8) |
| In-hospital mortality | 9 (18.0) | 4 (16.7) | 5 (19.2) |
| 30d overall mortality | 8 (16.0) | 4 (16.7) | 4 (15.4) |

*Legend:* ^†^Conversion to open surgery was defined as sternotomy and change to SAVR with a heart-lung-machine
