# Supplementary figures and images for: Transcatheter aortic valve replacement- management of patients with significant coronary artery disease undergoing aortic valve interventions: surgical compared to catheter-based approaches in hybrid procedures
Source: BMC Cardiovasc Disord. 2019 May 14;19:108. doi: 10.1186/s12872-019-1087-2 (PMC6515676; doi:10.1186/s12872-019-1087-2)

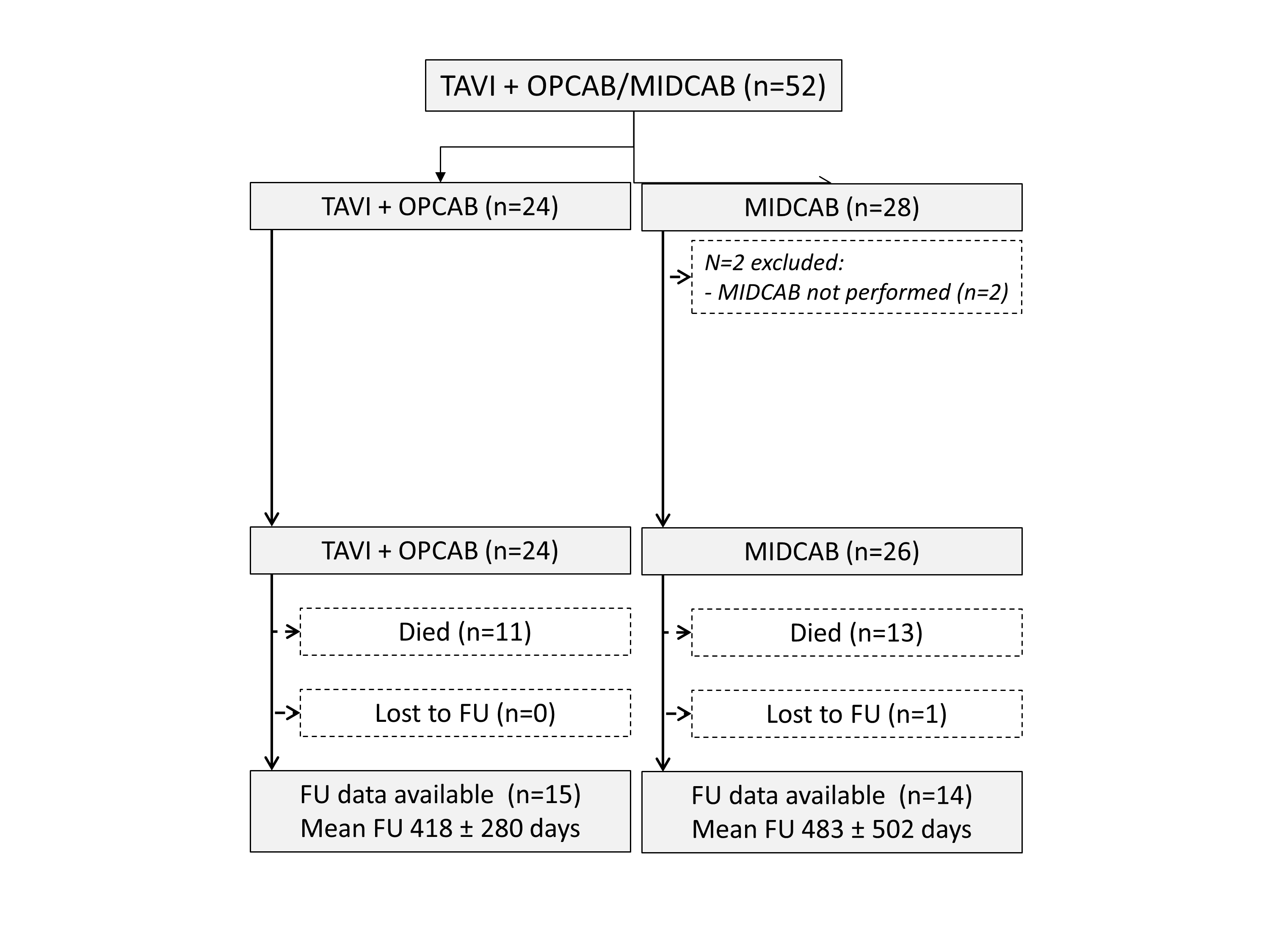

Supplement: Supplementary file 3 — Figure S1. Patient flow TAVR+OP vs. MIDCAB (TIF 528 kb) [file 12872_2019_1087_MOESM3_ESM.tif]

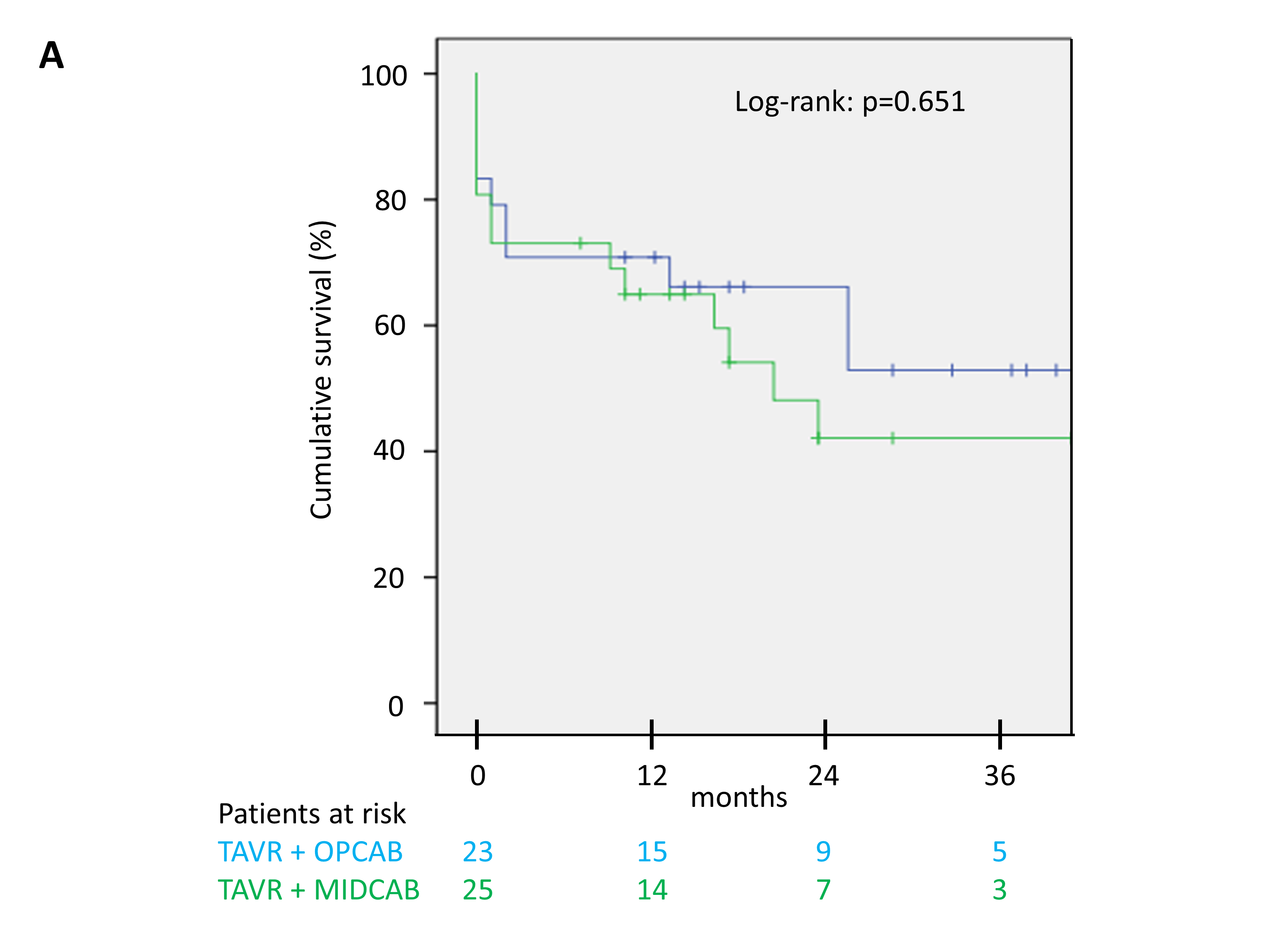

Supplement: Supplementary file 4 — Figure S2. Kaplan-Meier analysis for A) mortality and B) any rehospitalisation TAVR+OP vs. MIDCAB. (ZIP 461 kb) [file 12872_2019_1087_MOESM4_ESM.zip › Baumbach AS+CAD_20190403 Suppl 2A.tif]

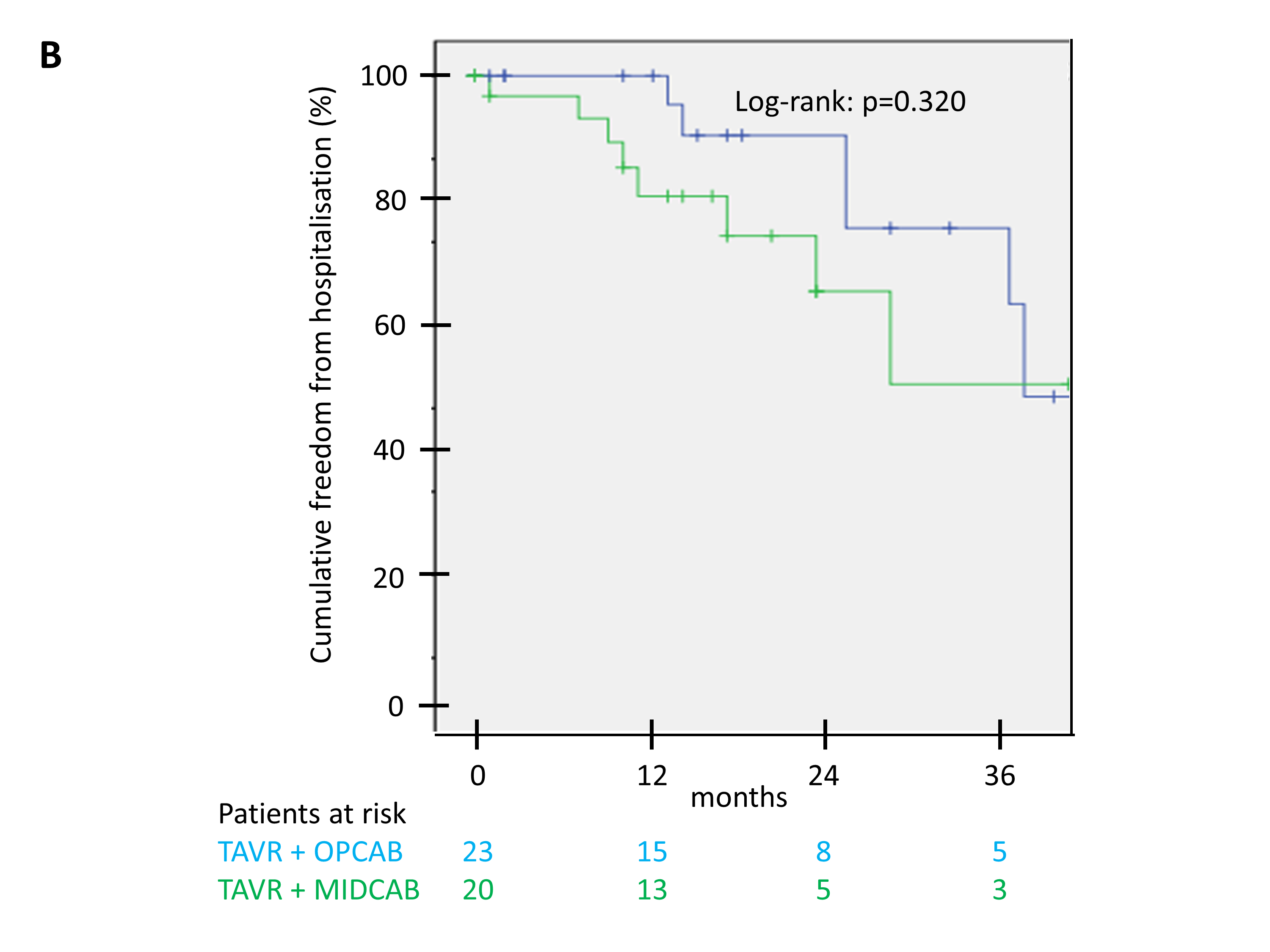

Supplement: Supplementary file 4 — Figure S2. Kaplan-Meier analysis for A) mortality and B) any rehospitalisation TAVR+OP vs. MIDCAB. (ZIP 461 kb) [file 12872_2019_1087_MOESM4_ESM.zip › Baumbach AS+CAD_20190403 Suppl 2B.tif]
